# Impact of the surgical modality for axillary lymph node dissection on postoperative drainage and seroma formation after total mastectomy

**DOI:** 10.1186/s13037-019-0199-z

**Published:** 2019-05-14

**Authors:** Hiroshi Isozaki, Yasuhisa Yamamoto, Shigeki Murakami, Sasau Matsumoto, Takehiro Takama

**Affiliations:** Department of Surgery, Oomoto Hospital, 1-1-5 Oomoto, Okayama, 700-0924 Japan

**Keywords:** Breast cancer, Total mastectomy, Axillary lymph node dissection, Sentinel node, Drain drainage, Seroma formation

## Abstract

**Background:**

The most common complications after total mastectomy with axillary lymph node treatment are prolonged drainage and seroma formation. The aim of this study was to find factors correlated with prolonged fluid discharge (prolonged drainage or seroma formation after 20th operative day or later), including surgical techniques or devices and clinical factors.

**Patients and methods:**

A total of 202 conclusive primary breast cancer patients underwent total mastectomy with axillary lymph node treatment between January 7, 2014 and June 20, 2018 at our hospital. The factors that correlated with the total fluid discharge volume and prolonged fluid discharge were examined statistically. The surgical modalities for total mastectomy with axillary treatment were classified into the following three groups:, Group A; skin flap formation by EC and axillary lymph node dissection by EC with ligation of the arteries and veins, Group B; skin flap formation by EC and axillary lymph node dissection by ultrasonic dissector (UD) without ligation of the vessels. Group D; skin flap formation by electrocautery (EC) and axillary lymph node dissection by picking up using forceps and ligation (PL).

**Results:**

The total fluid discharge volume and prolonged fluid discharge after total mastectomy with sentinel node retrieval (33 patients) were significantly lower than those after total mastectomy with axillary lymph node dissection (169 patients). In patients treated without drainage, a high rate of seroma formation and prolonged fluid discharge were observed, and 1 patient developed seroma infection.

In the total mastectomy with axillary lymph node dissection group, logistic regression analysis revealed that body mass index, 1-week drainage volume, and surgical modality were independently correlated with prolonged fluid discharge.

**Conclusions:**

The surgical procedure for axillary lymph node dissection should be considered to avoid prolonged fluid discharge, and the lymph vessels should be ligated in axillary lymph node dissection. An ultrasonic dissector was not effective in reducing the total fluid discharge volume. An optimal axillary lymph node dissection technique should be developed. For the patients without drainage, careful postoperative treatment should be given to avoid infectious seroma formation, even for patients who underwent total mastectomy with sentinel lymph node retrieval.

## Background

Axillary lymph node dissection remains an integral part of surgical treatment for patients with locally advanced breast cancer who undergo total mastectomy with axillary lymph node dissection for prognostic and curative purposes. Total mastectomy with sentinel node biopsy is employed for some patients. However, after total mastectomy with axillary lymph node dissection, long-term axillary drainage or seroma formation, which require frequent aspiration, are troublesome and delay chemotherapy. Seroma formation or prolonged drainage after total mastectomy with axillary lymph node dissection was reported to be related to age, breast size, tumor size, body mass index, axillary node status, surgical technique, surgical devices, mechanical or chemical obliteration of dead space, and active shoulder mobilization [1–4].

Regarding surgical devices, a number of studies on the use of an ultrasonic dissector (harmonic scalpel) or electrothermal bipolar vessel sealing system (Ligasure) have been reported for sealing lymphatic vessels without consistent results [5–13]. A randomized trial demonstrated that axillary dissection of lymph vessel ligation and dead space closure prevented seroma formation after total mastectomy with axillary lymph node dissection [14]. It was also reported that the surgical technique may affect the incidence of post-mastectomy seroma formation [15, 16].

In the present study, we analyzed the surgical modality of axillary lymph node treatment, as well as physiological or pathological factors in order to evaluate the causes of the increase in total drainage volume and prolonged fluid discharge, including seroma formation.

## Patients and methods

A total 613 breast cancer patients underwent surgery at Oomoto hospital (No. 0111442, medical corporation hospital, Okayama, Japan), between January 7, 2014 and June 20, 2018. Among them, 202 conclusive primary breast cancer patients who underwent total mastectomy with axillary lymph node treatment were enrolled in this study. Secondary breast cancer patients who underwent prior partial mastectomy of the ipsilateral breast or those undergoing simultaneous bilateral total mastectomy were excluded from the study.

The background of the patients of the present study is shown in Table 1. Of the 202 patients, 8 received preoperative chemotherapy.

**Table 1:** Demographic data of the breast cancer patients who underwent total mastectomy with axillary treatment

Number of patients		202
Age	Mean (sd) Years	63.1 (11.8)
	Median Years	65
Sex	Female	202
Body mass index	Mean (sd)	23.1 (4.0)
Hospital examination	Tumor palpation by patient	142
	Medical examination	43
	Others	17
Side (%)	Left	101
	Right	101
Number of tumors	Single	187
	Multiple	15
Subsites (%) (larger tumor if multiple)	1. Nipple	32
	2. Central portion	7
	3. Upper-inner quadrant	36
	4. Lower-outer quadrant	19
	5. Upper-outer quadrant	90
	6. Lower-outer quadrant	18
Tumor size (larger tumor if multiple)	Mean (sd) cm	2.9 (1.7)
Preoperative chemotherapy	No	194
	Yes	8

Regarding axillary lymph node treatment, sentinel lymph node retrieval was performed by the dye method. For the axillary lymph node dissection, the levels of axillary lymph node dissection are follows: Ax I indicates dissection of level I (low axilla), Ax II indicates dissection of level I and level II (mid axilla), and Ax III indicates dissection of level I, II, and III (apical axilla) according to the TNM classification [17].

**Fig. 1:**
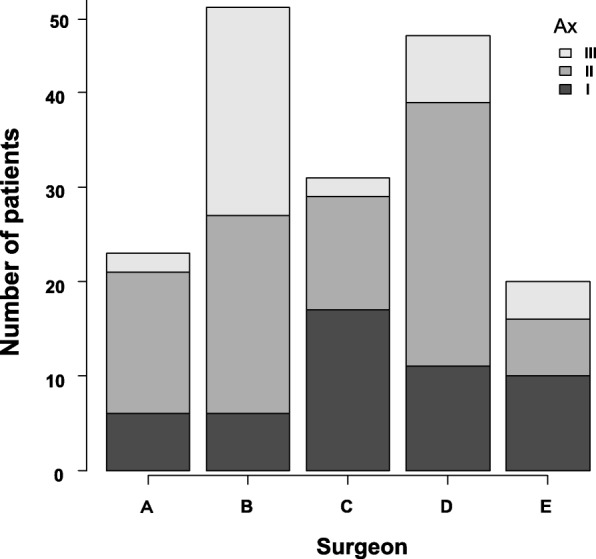
The number of patients and Ax levels for each surgeon

Five surgeons performed all of the operations. All surgeons performed mastectomy (skin flap formation) by electrocautery (EC). However, the techniques for axillary lymph node dissection differed by surgeon. Surgeons A, C, and E performed axillary lymph node dissection by EC with ligation of the arteries and veins. Surgeon B performed axillary lymph node dissection using an ultrasonic dissector (UD) without ligation of the vessels. Surgeon D performed axillary lymph node dissection by picking up the lymph nodes with fat tissue using forceps and ligated (PL) the connecting tissue, including lymph vessels, arteries, and veins. The number of patients and Ax levels for each surgeon are shown in Fig. 1. The long thoracic and thoracodorsal neurovascular bundles were preserved in all patients. Sentinel lymph node retrieval was performed via the same modality.

In this study, the surgical modality of total mastectomy with axillary treatment was classified into the following three groups:, Group A; skin flap formation by EC and axillary lymph node dissection by EC with ligation of the arteries and veins, Group B; skin flap formation by EC and axillary lymph node dissection by ultrasonic dissector (UD) without ligation of the vessels. Group D; skin flap formation by EC and axillary lymph node dissection by picking up using forceps and ligation (PL).

Excluding the 9 patients without drainage, one or two drain tubes were inserted, including into the axillary fossa. The number of drains was at the surgeon’s discretion. The drained fluid was collected in a negative pressure bag (suction reservoir with anti-reflex valve, TOKIBO, co., LTD.). The daily volumes of fluid were recorded by nurses. As a general rule, the drain was removed when the daily volume was reduced to 20 ml or less. Drains were removed by the respective surgeons, except for surgeon B (removed by surgeon A). All patients except those without drainage were hospitalized until the axillary drain was removed.

The relationship between the clinical factors and total fluid discharge volume was investigated. The total fluid discharge volume was the sum of all volumes discharged, including the aspirated volume of seroma after the removal of drains.

The end point of this study was prolonged fluid discharge. Prolonged fluid discharge was defined as prolonged drainage or seroma formation after the 20th operative day or later.

Univariate analysis was performed between the total mastectomy with sentinel node retrieval group and total mastectomy with axillary lymph node dissection group. In the total mastectomy with axillary lymph node dissection group, univariate and multivariate analyses were performed in relation to prolonged fluid discharge.

### Statistical analysis

All statistical analyses were performed using EZR (Saitama Medical center, Jichi Medical University, Saitama, Japan), which is a graphical user interface for R (The R Foundation for Statistical Computing, Vienna, Austria). More precisely, it is a modified version of R commander designed to add statistical functions frequently used in biostatistics [18]. In general, *p*-values < 0.05 by the unpaired *t*-test, one-way ANOVA, Pearson product-moment correlation coefficient or logistic regression were considered significant.

## Results

Clinicopathological factors of total mastectomy with sentinel node retrieval and total mastectomy and with axillary lymph node dissection (Ax I, II, and III) (Table 2).

**Table 2:** Clinicopathological factors of patient who underwent total mastectomy with sentinel node retrieval or total mastectomy with axillary lymph node dissection (Ax I, II, and III)

Factor	Group	Total mastectomy Sentinel node retrieval	Total mastectomy Axillary lymph node dissection	*p*-value
Number of patients		33	169	
		mean (sd)	mean (sd)	
Age mean (sd) years		69.6 (11.8)	61.9 (11.4)	0.05
Body mass index		22.3 (3.1)	23.2 (4.1)	
Side of the tumor (%)	Left	13 (39.4)	78 (46.2)	< 0.001
	Right	20 (60.6)	91 (53.8)	0.019
Preoperative chemotherapy (%)	No	33 (100.0)	161 (95.3)	0.013
	Yes	0 (0.0)	8 (4.7)	0.057
Number of lymph nodes retrieved		2.91 (1.59)	14.28 (7.13)	0.302
Number of metastatic lymph nodes		0.27 (0.63)	1.86 (3.86)	< 0.001
Weight of specimen		344.8 (159.9)	449.3 (228.6)	< 0.001
Size of tumor		24.2 (17.4)	30.4 (16.8)	0.057
Stage (TMN)	1	16 (48.5)	60 (35.5)	0.534
	2	16 (48.5)	94 (55.6)	
	3	1 (3.0)	13 (7.7)	
	4	0 (0.0)	2 (1.2)	
Drainage	No	9 (27.3)	0 (0.0)	< 0.001
	Yes	24 (72.7)	169 (100.0)	
Volume of drainage during 1 week		312.1 (153.4)	588.3 (279.4)	< 0.001
Total fluid discharge volume		333.1 (262.1)	1456.3 (817.4)	< 0.001
Drainage volume the day before drain removal		15.50 (9.10)	19.05 (9.61)	0.05
Post-operative day of drain removal		8.4 (4.23)	14.2 (6.24)	< 0.001
Seroma formation	No	27 (81.8)	136 (80.5)	1
	Yes	6 (18.2)	33 (19.5)	
Number	0	26 (78.8)	136 (80.5)	
	1	0 (0.0)	19 (11.2)	
	2	3 (9.1)	6 (3.6)	
	3	3 (9.1)	3 (1.8)	
	4	1 (3.0)	4 (2.4)	
	6	0 (0.0)	1 (0.6)	
Surgical modality				
Group D		7 (21.2)	46 (27.2)	0.21
Group A		11 (33.3)	74 (43.8)	
Group B		15 (45.5)	49 (29.0)	
Prolonged fluid discharge	No	29 (87.9)	117 (69.2)	0.033
	Yes	4 (12.1)	52 (30.8)	
Hospital stay	Days	14.0 (5.1)	22.3 (7.2)	< 0.001
Complications				
seroma infection extirpation of seroma by surgery (re-drainage)		1	0	
re-drainage		0	2	
infection		0	2	
bleeding		0	1	
flap necrosis		0	0	
Lymph edema of upper limbs (%)	No	33 (100.0)	149 (88.2)	0.05
	Yes	0 (0.0)	20 (11.8)	

Prolonged fluid discharge: prolonged drainage or seroma formation after the 20th operative day or later

Group D: skin flap formation by electrocautery and axillary lymph node dissection by picking up using forceps and ligation (Surgeon D)

Group A: skin flap formation by electrocautery and axillary lymph node dissection by electrocautery with ligation of the arteries and veins (Surgeons A, C and E)

Group B: skin flap formation by electrocautery and axillary lymph node dissection by ultrasonic dissector without ligating the vessels (Surgeon B)

Of the 202 patients who underwent total mastectomy with axillary treatment, 33 underwent total mastectomy with sentinel node retrieval and 169 underwent total mastectomy with axillary lymph node dissection. The total volume of drainage (333 ml) and incidence (12%) of prolonged fluid discharge after total mastectomy with sentinel node retrieval were significantly lower than those (1456 ml, 31%) after total mastectomy with axillary lymph node dissection. Among the 33 patients in the total mastectomy with sentinel node retrieval group, 9 had no drainage, 6 had seroma formation, 3 patients developed prolonged fluid discharge, and 1 patient had seroma infection requiring surgical treatment (resection of seroma) with re-drainage.

Clinicopathological factors related to prolonged fluid discharge after total mastectomy with axillary lymph node dissection (Table 3).

**Table 3:** Clinicopathological factors related to prolonged fluid discharge after total mastectomy with axillary lymph node dissection

Factor	Group	No	Yes	*p*-value
Number of patients		117	52	
		mean (sd)	mean (sd)	
Age		61.6 (11.8)	62.5 (10.9)	0.636
Body mass index		22.6 (3.82)	24.6 (4.6)	0.005
Preoperative chemotherapy	No	110 (94.0)	51 (98.1)	0.437
	Yes	7 (6.0)	1 (1.9)	
Site of breast tumor (%)	Right	63 (53.8)	28 (53.8)	1
	Left	54 (46.2)	24 (46.2)	
Number of tumors	Multiple	8 (6.8)	6 (11.5)	0.366
	Single	109 (93.2)	46 (88.5)	
Weight of specimen		410.9 (196.9)	535.7 (270.0)	0.001
Size		30.9 (17.3)	30.6 (16.1)	0.892
Level of axillary lymph node dissection (%)	Ax I	40 (34.2)	10 (19.2)	0.066
	Ax II	56 (47.9)	26 (50.0)	
	Ax III	21 (17.9)	16 (30.8)	
Number of lymph nodes retrieved		14.8 (7.4)	14.5 (6.4)	0.77
Number of metastatic lymph nodes		1.6 (3.5)	2.4 (4.5)	0.226
Volume of drainage during 1 week		506.0 (244.9)	773.7 (265.0)	< 0.001
Total fluid discharge volume		1161.7 (615.1)	2048.0 (794.8)	< 0.001
Drainage volume the day before drain removal		17.2 (8.3)	23.2 (11.1)	< 0.001
seroma formation (%)	No	117 (100.0)	19 (36.5)	< 0.001
	Yes	0 (0.0)	33 (63.5)	
Re-drainage (%)	No	117 (100.0)	50 (96.2)	0.093
	Yes	0 (0.0)	2 (3.8)	
Lymph edema of the upper limbs (%)	No	102 (87.2)	47 (90.4)	0.617
	Yes	15 (12.8)	5 (9.6)	
Surgical modality	Group D	41 (35.0)	5 (9.6)	0.001
	Group A	48 (41.0)	26 (50.0)	
	Group B	28 (23.9)	21 (40.4)	

No: no prolonged drainage or seroma formation after the 20th operative day or later

Yes: prolonged drainage or seroma formation after the 20th operative day or later

Group D: skin flap formation by electrocautery and axillary lymph node dissection by picking up using forceps and ligation (Surgeon D)

Group A: skin flap formation by electrocautery and axillary lymph node dissection by electrocautery with ligation of the arteries and veins (Surgeons A, C and E)

Group B: skin flap formation by electrocautery and axillary lymph node dissection by ultrasonic dissector without ligating the vessels (Surgeon B)

**Fig. 2:**
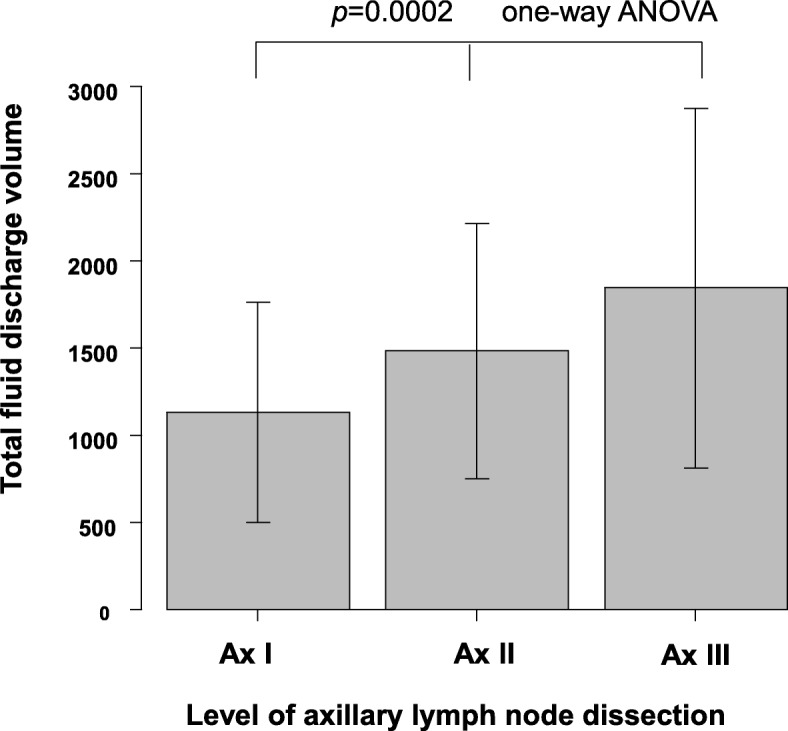
The relationship between the level of axillary lymph node dissection and total fluid discharge volume

**Fig. 3:**
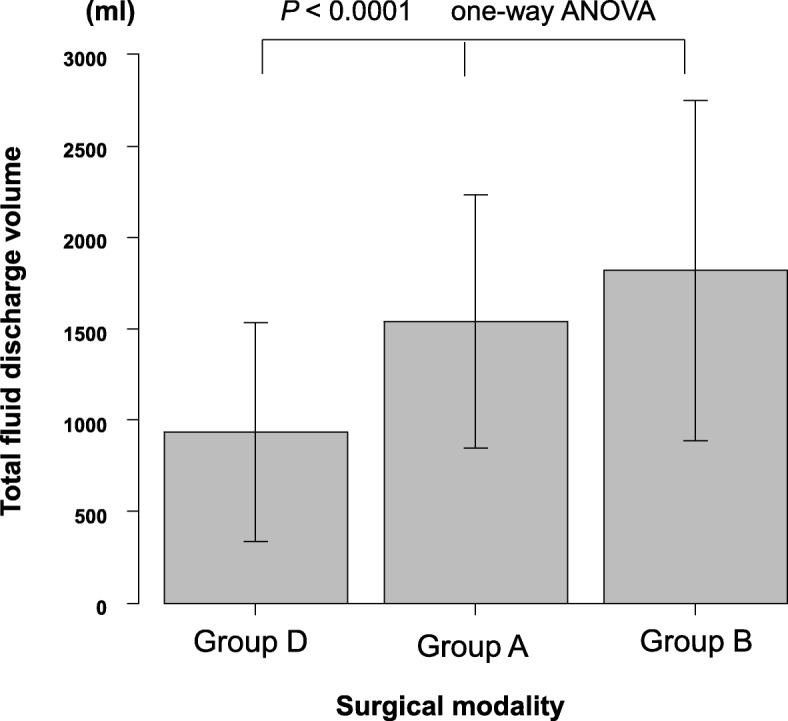
The relationship between the surgical modality and total fluid discharge volume. Group D: skin flap formation by electrocautery and axillary lymph node dissection by picking up using forceps and ligation (Surgeon D). Group A: skin flap formation by electrocautery and axillary lymph node dissection by electrocautery with ligation of the arteries and veins (Surgeons A, C and E). Group B: skin flap formation by electrocautery and axillary lymph node dissection by ultrasonic dissector without ligating the vessels (Surgeon B)

The total fluid discharge volume according to the level of axillary lymph node dissection is shown in Fig. 2. The total fluid discharge volume based on the surgical modality is shown in Fig. 3.

Based on prolonged fluid discharge, univariate analysis revealed the following factors to be significantly related to prolonged fluid discharge after total mastectomy with axillary lymph node dissection: body mass index, weight of specimen, level of axillary lymph node dissection, volume of drainage during 1 week, total fluid discharge volume, drainage volume the day before drain removal, seroma formation, and surgical modality (Table 3).

The logistic regression analysis demonstrated body mass index, drainage volume during 1 week, and surgical modality to be independently correlated with prolonged fluid discharge (Table 4).

**Table 4:** Logistic regression in relation to prolonged fluid discharge after total mastectomy with axillary lymph node dissection

Factor	Odds ratio	*p*-value *<0.05
Body mass index < 25 vs ≧25	2.54 (1.08–5.96)	0.033*
Specimen weight < 400 g vs ≧400 g	0.83 (0.35–1.93)	0.66
Tumor size < 30 mm vs ≧30 mm	0.75 (0.34–1.65)	0.47
Level of axillary lymph node dissection Ax I vs II vs III	1.34 (0.72–2.48)	0.35
Number of axillary lymph nodes dissected < 15 vs ≧15	1.02 (0.46–2.27)	0.97
Number of metastatic lymph nodes = 0 vs ≧1	1.11 (0.48–2.58)	0.81
Drainage volume during 1 week < 500 ml vs ≧500 ml	4.25 (1.55–11.60)	0.005*
Surgical modality: Group D vs Group A vs Group B	1.86 (1.06–3.26)	0.03*

Prolonged fluid discharge: prolonged drainage or seroma formation after the 20th operative day or later

Group D: skin flap formation by electrocautery and axillary lymph node dissection by picking up using forceps and ligation (Surgeon D)

Group A: skin flap formation by electrocautery and axillary lymph node dissection by electrocautery with ligation of the arteries and veins (Surgeons A, C and E)

Group B: skin flap formation by electrocautery and axillary lymph node dissection by ultrasonic dissector without ligating the vessels (Surgeon B)

## Discussion

In the present retrospective study, we investigated patients who underwent total mastectomy with sentinel node retrieval and those who underwent total mastectomy with axillary lymph node dissection. As a result, the total volume of drainage and incidence of prolonged fluid discharge after total mastectomy with sentinel node retrieval were significantly lower than those after total mastectomy with axillary lymph node dissection. However, among 9 patients with no drainage, 6 patients had seroma formation, 3 patients developed prolonged fluid discharge (20th postoperative day or longer), and 1 patient with a high body mass index (29%) underwent surgery again because of infectious seroma. Consequently, careful monitoring for infectious seroma formation is needed for breast cancer patients undergoing total mastectomy with axillary treatment without drainage [19].

Regarding total mastectomy with axillary lymph node dissection, many factors have been reported to be related to marked postoperative fluid discharge from drains or seroma formation. In the present study, the univariate analysis and Pearson product-moment correlation coefficients demonstrated that body mass index, specimen weight, tumor size, level of axillary lymph node dissection, number of lymph nodes dissected, metastatic lymph nodes, and 1-week drainage volume were significantly correlated with total fluid discharge (data not shown), consistent with previous reports. The previous study noted a large total drainage volume (3300–4500 ml) after total mastectomy with axillary lymph node dissection by electrocautery [9, 16]. The total drainage volume in this study (1434 ml) was lower than that in the previous report. In this study, the drains were removed when the daily volume decreased to 20 ml or less. As there is no consensus on the time of removal, some drains are removed when the drainage volume is less than 30 ml [9, 20], whereas others were removed at less than 50 ml [19, 21]. The mean postoperative day of drain removal in this study (14.0 day) was similar to that in previous reports (17.9 day) in which the drain was removed at a volume less than 30 ml [9]. Although early drain removal was proposed, a previous study recommended long-term axillary drainage after total mastectomy with axillary lymph node dissection because the highest incidence of seroma and largest aspiration volumes were found in patients with short-term drainage after total mastectomy with axillary lymph node dissection [22].

According to the logistic regression analysis of independent factors for prolonged fluid discharge, the surgical modality, body mass index, and drainage volume during 1 week are significant independent factors. This retrospective case control study also examined the surgical procedures. All five surgeons performed mastectomy (skin flap formation) using electrocautery, but for axillary lymph node dissection, individual surgeons employed different procedures. Surgeons A, C, and E performed axillary lymph node dissection using electrocautery. Surgeon B used an ultrasonic dissector for en-bloc axillary lymph node dissection. Surgeon B performed Ax III in more patients than the other surgeons, resulting in the largest total drainage volume (not significantly different from surgeon A or C. Moreover, the use of an ultrasonic dissector for axillary lymph node dissection did not reduce the total drainage volume. Surgeon D performed axillary lymph node dissection by picking up lymph nodes and fat tissue using forceps, and finely ligating all connecting tissue and vessels (lymph vessels, arteries, and veins). As a result, the total fluid discharge volume of surgeon D was significantly lower than that of the other surgeons. Therefore, we consider postoperative fluid collection after total mastectomy with axillary lymph node dissection to be mainly caused by disrupted axillary lymphatics rather than serous fluid formation from mastectomy flaps.

As for the ultrasonic dissector (harmonic scalpel), many reports have stated that harmonic scalpel dissection is advantageous for decreasing postoperative drainage and seroma formation after total mastectomy with axillary lymph node dissection [5–9]. However, in these reports, all surgical procedures (skin flap and axillary dissection) employed the harmonic scalpel. Thus, the advantage of the ultrasonic dissector for only axillary lymph node dissection remains unclear. One study from India [11] in which a surgical protocol similar to ours was employed reported no significant differences between the two groups.

Regarding the surgical procedure, a randomized study found that the group in which all of the tissue connecting the axillary vein bundle to the specimen was ligated (ligating lymph vessels), the anterior edge of the latissimus dorsi was sutured to the chest wall, and the skin flap was fixed to the underlying muscle had a shorter drainage duration and lower incidence of seroma formation than the group in which electrocautery was used [14]. Although three surgical elements were included, which makes interpretation difficult, ligation of lymph vessels was found to play an important role.

In summary, the surgical modality, body mass index, and drainage volume during 1 week were demonstrated to be independent factors correlated with prolonged fluid discharge. Therefore, the surgical technique and axillary lymph node dissection should be carefully considered for the treatment of breast cancer patients scheduled for total mastectomy with axillary lymph node dissection. Namely, the lymph vessels should be ligated during axillary lymph node dissection. Furthermore, the ultrasonic dissector was not effective in reducing the total drainage volume in the present study.

Although the previous report [23] found a strong correlation between the total hospital drainage and the frequency of seroma and upper extremity edema, in the present study, the frequency of lymphedema of the upper limbs in the prolonged fluid discharge group (9.6%) was not different from that in the non-prolonged fluid discharge group (12.8%).

This case control study has some limitations. The number of patients was relatively small. Although the drains were removed when the drainage volume within the previous 24 h decreased to 20 ml or less, as a general rule, the decision was mainly made by the chief surgeon, with little difference in the timing. This is the first analysis of total fluid discharge volume or prolonged drainage and seroma formation at our hospital. This study was started when surgeon B (a specialist in digestive surgery who used an ultrasonic dissector) began performing breast cancer surgery. As such, it may be referred to as a past cohort study. In the future, a multicenter prospective study should be carried out to investigate the effectiveness of surgical procedures, including surgical devices. Regarding the surgical procedure of axillary lymph node dissection, from an oncological point of view, lymph nodes should be removed as a lump with neighboring adipose tissue (en-bloc dissection). Although sentinel node biopsy is widely accepted in the surgical treatment of breast cancer, axillary lymph node dissection by picking up the lymph nodes with fat tissue using forceps should be discussed based on curability. During axillary lymph node dissection, in order to avoid marked postoperative lymphorrhea [22], the location to ligate the lymphatic tract is important. Thus, an optimal axillary lymph node dissection technique should be developed.

## Conclusions

The surgical procedure for axillary lymph node dissection should be considered to avoid prolonged fluid discharge, and the lymph vessels should be ligated during axillary lymph node dissection. An ultrasonic dissector was not effective in reducing the total fluid discharge volume. An optimal axillary lymph node dissection technique should be developed.

In the patients with no drainage, careful postoperative treatment should be taken to avoid infectious seroma formation, even for those who underwent total mastectomy with sentinel lymph node retrieval.

## Abbreviations

Ax I: Level I
Ax II: Level II
Ax III: Level III according to the TNM classification
Ax: Axillary lymph node dissection
EC: Electrocautery
PL: Picking up using forceps and ligation
UD: Ultrasonic dissector

## References

[1] Srivastava V, Basu S, Shukla VK (2012). Seroma formation after breast cancer surgery: what we have learned in the last two decades. J Breast Cancer.

[2] Lin YP, Yin WJ, Yan TT, Zhou LH, GH DI, Wu J (2011). Risk factors for postoperative seromas in Chinese breast cancer patients. Chin Med J.

[3] Zieliński J, Jaworski R, Irga N, Kruszewski JW, Jaskiewicz J (2013). Analysis of selected factors influencing seroma formation in breast cancer patients undergoing mastectomy. Arch Med Sci.

[4] Abe M, Iwase T, Takeuchi T, Murai H, Miura S (1998). A Randomized Controlled Trial on the Prevention of Seroma after Partial or Total Mastectomy and Axillary Lymph Node Dissection. Breast Cancer.

[5] Huang Jinbo, Yu Yinghua, Wei Changyuan, Qin Qinghong, Mo Qinguo, Yang Weiping (2015). Harmonic Scalpel versus Electrocautery Dissection in Modified Radical Mastectomy for Breast Cancer: A Meta-Analysis. PLOS ONE.

[6] Iovino F, Auriemma PP, Ferraraccio F, Antoniol G, Barbarisi A (2012). Preventing seroma formation after axillary dissection for breast cancer: a randomized clinical trial. Am J Surg.

[7] Khan S, Khan S, Chawla T, Murtaza G (2014). Harmonic scalpel versus electrocautery dissection in modified radical mastectomy: a randomized controlled trial. Ann Surg Oncol.

[8] Archana A, Sureshkumar S, Vijayakumar C, Palanivel C (2018). Comparing the harmonic scalpel with electrocautery in reducing postoperative flap necrosis and seroma formation after modified radical mastectomy in carcinoma breast patients: a double-blind prospective randomized Control Trail. Cureus..

[9] Faisal M, Fathy H, Shaban H, Abuelela ST, Marie A, Khaled I. A novel technique of harmonic tissue dissection reduces seroma formation after modified radical mastectomy compared to conventional electrocautery: a single-blind randomized controlled trial. Patient Saf Surg. 2018. 10.1186/s13037-018-0155-3. 10.1186/s13037-018-0155-3 PMC5956966 29796089

[10] Kontos M, Kothari A, Hamed H (2008). Effect of harmonic scalpel on seroma formation following surgery for breast cancer: a prospective randomized study. J BUON.

[11] Shanmugam S, Govindasamy G, Hussain SA, Rao PSH (2017). Axillary dissection for breast cancer using electrocautery versus ultrasonic dissectors: a prospective randomized study. Indian J Cancer.

[12] Manouras A, Markogiannakis H, Genetzakis M, Filippakis GM, Lagoudianakis EE, Kafiri G (2008). Modified radical mastectomy with axillary dissection using the electrothermal bipolar vessel sealing system. Arch Surg.

[13] Antonio M, Pietra T, Domenico L, Massimo D, Ignazio R, Antonio N, et al. Does LigaSure reduce fluid drainage in axillary dissection? A randomized prospective clinical trial. Ecancermedicalscience. 2007. 10.3332/eCMS.2007.61. 10.3332/eCMS.2007.61 PMC3223974 22275958

[14] Gong Y, Xu J, Shao J, Cheng H, Wu X, Zhao D (2010). Prevention of seroma formation after mastectomy and axillary dissection by lymph vessel ligation and dead space closure: a randomized trial. Am J Surg.

[15] van Bemmel AJ, van de Velde CJ, Schmitz RF, Liefers GJ (2011). Prevention of seroma formation after axillary dissection in breast cancer: a systematic review. Eur J Surg Oncol.

[16] Faisal M, Abu-Elela ST, Mostafa W, Antar O. Efficacy of axillary exclusion on seroma formation after modified radical mastectomy. World J Surg Oncol. 2016. 10.1186/s12957-016-0801-0. 10.1186/s12957-016-0801-0 PMC4761189 26897384

[17] UICC (Union for International Cancer Control) (2017). TNM classification of malignant tumors.

[18] Kanda Y (2013). Investigation of the freely available easy-to-use software ‘EZR’ for medical statistics. Bone Marrow Transplant.

[19] Stoyanov GS, Tsocheva D, Marinova K, Dobrev E, Nenkov R. Drainage after modified radical mastectomy - a methodological mini-review. Cureus. 2017. 10.7759/cureus.1454. 10.7759/cureus.1454 PMC5590707 28929038

[20] Barton A, Blitz M, Callahan D, Yakimets W, Adams D, Dabbs K (2006). Early removal of postmastectomy drains is not beneficial: results from a halted randomized controlled trial. Am J Surg.

[21] Okada N, Narita Y, Takada M, Kato H, Ambo Y, Nakamura F (2015). Early removal of drains and the incidence of seroma after breast surgery. Breast Cancer.

[22] Andeweg CS, Schriek MJ, Heisterkamp J, Roukema JA (2011). Seroma formation in two cohorts after axillary lymph node dissection in breast cancer surgery: does timing of drain removal matter?. Breast J.

[23] Tadych K, Donegan WL (1987). Postmastectomy seromas and wound drainage. Surg Gynecol Obstet.

